# Origin-independent plasmid replication occurs in vaccinia virus cytoplasmic factories and requires all five known poxvirus replication factors

**DOI:** 10.1186/1743-422X-2-23

**Published:** 2005-03-22

**Authors:** Frank S De Silva, Bernard Moss

**Affiliations:** 1 Laboratory of Viral Diseases, National Institute of Allergy and Infectious Diseases, National Institutes of Health, Bethesda, Maryland 20892-0445, USA

## Abstract

**Background:**

Replication of the vaccinia virus genome occurs in cytoplasmic factory areas and is dependent on the virus-encoded DNA polymerase and at least four additional viral proteins. DNA synthesis appears to start near the ends of the genome, but specific origin sequences have not been defined. Surprisingly, transfected circular DNA lacking specific viral sequences is also replicated in poxvirus-infected cells. Origin-independent plasmid replication depends on the viral DNA polymerase, but neither the number of additional viral proteins nor the site of replication has been determined.

**Results:**

Using a novel real-time polymerase chain reaction assay, we detected a >400-fold increase in newly replicated plasmid in cells infected with vaccinia virus. Studies with conditional lethal mutants of vaccinia virus indicated that each of the five proteins known to be required for viral genome replication was also required for plasmid replication. The intracellular site of replication was determined using a plasmid containing 256 repeats of the *Escherichia coli lac *operator and staining with an *E. coli lac *repressor-maltose binding fusion protein followed by an antibody to the maltose binding protein. The *lac *operator plasmid was localized in cytoplasmic viral factories delineated by DNA staining and binding of antibody to the viral uracil DNA glycosylase, an essential replication protein. In addition, replication of the *lac *operator plasmid was visualized continuously in living cells infected with a recombinant vaccinia virus that expresses the *lac *repressor fused to enhanced green fluorescent protein. Discrete cytoplasmic fluorescence was detected in cytoplasmic juxtanuclear sites at 6 h after infection and the area and intensity of fluorescence increased over the next several hours.

**Conclusion:**

Replication of a circular plasmid lacking specific poxvirus DNA sequences mimics viral genome replication by occurring in cytoplasmic viral factories and requiring all five known viral replication proteins. Therefore, small plasmids may be used as surrogates for the large poxvirus genome to study *trans*-acting factors and mechanism of viral DNA replication.

## Background

Vaccinia virus (VAC), the prototype for the family *Poxviridae*, is a large double-stranded DNA virus that encodes numerous enzymes and factors needed for RNA and DNA synthesis, enabling it to replicate in the cytoplasm of infected cells [1]. More than 20 viral proteins including a multi-subunit RNA polymerase and stage specific transcription factors are involved in viral RNA synthesis [2]. Genetic and biochemical studies identified five viral proteins essential for viral DNA replication, namely the viral DNA polymerase [3-8], polymerase processivity factor [9,10], DNA-independent nucleoside triphosphatase [11-13], serine/threonine protein kinase [14-17], and uracil DNA glycosylase [18-21]. In addition, the virus encoded Holliday junction endonuclease is required for the resolution of DNA concatemers into unit-length genomes [22]. Other proteins that may contribute to viral DNA replication, include DNA type I topoisomerase, single stranded DNA binding protein, DNA ligase, thymidine kinase, thymidylate kinase, ribonucleotide reductase and dUTPase (reviewed in reference [1]).

The VAC genome consists of a 192 kbp linear duplex DNA with covalently closed hairpin termini [23,24]. A model for poxvirus DNA replication begins with the introduction of a nick near one or both ends of the hairpin termini, followed by polymerization of nucleotides at the free 3'-OH end, strand displacement and concatemer resolution [25,26]. Nicking is supported by changes in the sedimentation of the parental DNA following infection, and labeling studies suggested that replication begins near the ends of the genome [27,28]. Efforts to locate a specific origin of replication in the VAC genome led to the surprising conclusion that any circular DNA replicated as head-to-tail tandem arrays in cells infected with VAC [29,30]. Origin-independent plasmid replication was also shown to occur in the cytoplasm of cells infected with other poxviruses including Shope fibroma virus and myxoma virus as well as with African swine fever virus [30,31]. In contrast, studies with linear minichromosomes containing hairpin termini provided evidence for *cis*-acting elements in VAC DNA replication [32]. It was considered that plasmid replication might be initiating non-specifically, perhaps at random nicks in DNA.

Although transfected plasmids were used to study the resolution of poxvirus concatemer junctions [33-37], the system has not been exploited for studies of viral DNA synthesis. The goal of the present study was to determine how closely plasmid replication mimics viral genome replication. For example, if some viral proteins are needed for initiating DNA synthesis at specific origins near the ends of the viral genome, they might not be required for plasmid replication. In addition, we were curious as to whether synthesis of plasmid DNA occurs diffusely in the cytoplasm, since the transfected DNA enters cells independently of virus and contains no viral targeting sequences. Contrary to these speculations, we found that each of the five viral proteins known to be required for viral genome replication was needed for origin-independent replication of plasmids. Moreover, both plasmid and genome replication occurred in discrete viral cytoplasmic factory areas. Thus, small circular plasmids are useful surrogates for the large viral genome in studying the mechanism of poxvirus DNA replication and the *trans*-acting factors required.

## Results

### Determination of plasmid replication by real-time PCR

**Figure 1:**
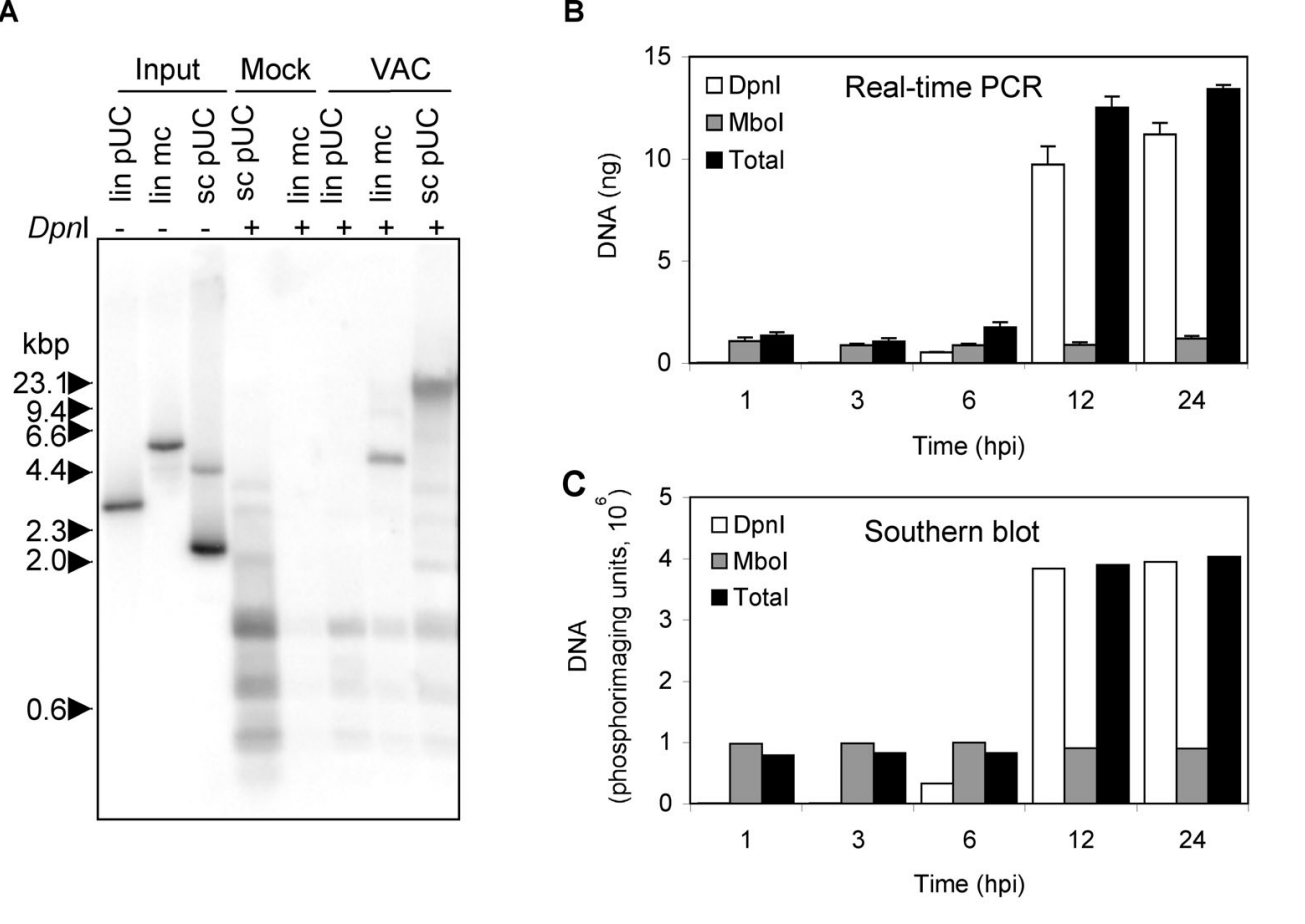
Replication of transfected DNA in VAC-infected cells. **(A) **Southern blot of replicated circular plasmid and linear minichromosome. B-SC-1 cells were infected with VAC and 1 h later transfected with equal molar amounts (20 fmol) of super coiled pUC13 (sc pUC), pUC13 linearized by digestion with *EcoR*I (lin pUC), linear minichromosome containing pUC13 and 1.3 kbp viral telomeric sequences (lin mc). As a control, cells were mock infected and transfected with 20 fmol of linear minichromosome or 10 times that amount (200 fmol) of super coiled pUC13. At 24 h after infection or mock infection, cells were collected and total DNA extracted. Total DNA (2 μg) was digested with *Dpn*I subjected to agarose gel electrophoresis and analyzed by Southern blot hybridization using a ^32^P-labeled pUC13 probe. Samples (0.5 fmol of lin pUC, 0.5 fmol of lin mc, 1 fmol sc pUC) of the DNA used for transfections (input DNA) were also analyzed. The positions of marker DNA (kbp) are shown on the left. **(B) **Real-time PCR of replicated plasmid. BS-C-1 cells were transfected with the plasmid p716 at 24 h prior to infection with VAC. At indicated hours post infection (hpi), cells were harvested and total DNA extracted. DNA was untreated or treated with *Dpn*I or *Mbo*I and analyzed by real-time PCR using primers specific to plasmid DNA. **(C) **Quantification of Southern blot. DNA described in panel (B) was digested with *EcoR*I prior to *Mbo*I or *Dpn*I treatment. The digested DNA samples were subjected to gel electrophoresis, transferred to a Nylon membrane, hybridized to a ^32^P-labeled p716 probe, and the radioactivity quantified with a phosphoImager.

The replication of plasmids and linear minichromosomes, which were transfected into cells infected with VAC, was previously demonstrated by autoradiography following hybridization of ^32^P-labeled probes to Southern blots [29,30,32]. Methylated input DNA prepared in *E. coli *was distinguished from unmethylated DNA replicated in infected mammalian cells by digestion with *Dpn*I and *Mbo*I, which cleave G^m^ATC and GATC sequences, respectively. DeLange and McFadden [30] had reported an 8-fold net increase of a circular plasmid lacking viral sequences in rabbit cells infected with myxoma virus, whereas Du and Traktman [32] had seen a 2-fold net increase of a linear minichromosome containing VAC genome termini in mouse L cells infected with VAC, but a much lower increase of a circular plasmid lacking viral sequences. We compared the replication of three types of DNA (super coiled circular, linear, and linear minichromosome) in African green monkey BS-C-1 cells, which has become a standard cell line for VAC research. Southern blot analysis of the *Dpn*I-digestion products of DNA isolated from cells infected with VAC and transfected with super coiled pUC13 revealed a prominent high molecular weight band migrating above the 23.1-kbp marker, presumably representing head-to-tail concatemers (Fig. 1A). A prominent *DpnI*-resistant band, migrating between the 4.4 and 6.6 kbp markers, was obtained by digestion of DNA from infected BS-C-1 cells transfected with the covalently closed minichromosome. However, only small digestion products were obtained upon *Dpn*I-treatment of DNA from cells transfected with linear pUC13. In addition, *Dpn*I-resistant bands were not detected by digestion of DNA from mock-infected cells transfected with a linear minichromosome or 10 times more super coiled plasmid (Fig. 1A). This experiment confirmed the need for VAC infection and either a circular plasmid or a linear minichromosome template for DNA replication. Moreover, we did not see greater replication of the linear minichromosome than the circular plasmid as had been reported (32).

To improve quantification of plasmid replication and to establish a non-radioactive method for rapid analysis of multiple samples, we devised a real-time PCR assay using primers 152 bp apart that flanked two *Dpn*I/*Mbo*I sites in a circular plasmid lacking VAC DNA sequences. In initial experiments, we followed the protocol of previous studies by transfecting the plasmid after infection [29,30]. However, *Mbo*I-resistant input DNA as well as *Dpn*I-resistant replicated DNA increased with time, suggesting that entry of DNA into the cell occurred continuously even though the medium was changed at 4 h (data not shown). To avoid this problem in subsequent experiments, DNA was transfected 24 h prior to infection. Total DNA was isolated at various times, digested with *Dpn*I, *Mbo*I, or left uncut and subjected to real-time PCR. Under these conditions, *Mbo*I-resistant DNA did not increase, whereas *Dpn*I-resistant DNA increased ~18 fold between 3 and 6 h and ~400 fold by 24 h (Fig. 1B). Moreover, total DNA increased ~10 fold. Increased *Dpn*I-resistant DNA was not detected in mock-infected cells (data not shown).

Previous Southern blotting studies had indicated that plasmid replication paralleled genome replication [30]. We compared the kinetics of plasmid replication obtained by real-time PCR with Southern blotting. For the latter analysis, total DNA was first digested with *EcoR*I to resolve head-to-tail concatemers into linear units followed by digestion with *Mbo*I or *Dpn*I. After electrophoresis, the DNA was transferred to a nylon membrane, hybridized to a ^32^P-labeled plasmid probe, and the amount of DNA quantified using a PhosphorImager. The *Dpn*I-resistant and total DNA increased with time, whereas the *Mbo*I-resistant DNA did not (Fig. 1C). The Southern blot analysis suggested that the amount of replicated plasmid plateaued after 12 h, whereas it continued to increase slightly as determined by PCR (Fig. 1B), suggesting that the latter method has the greater dynamic range as well as being more convenient.

### Determination of the trans-acting factors required for plasmid replication

**Figure 2:**
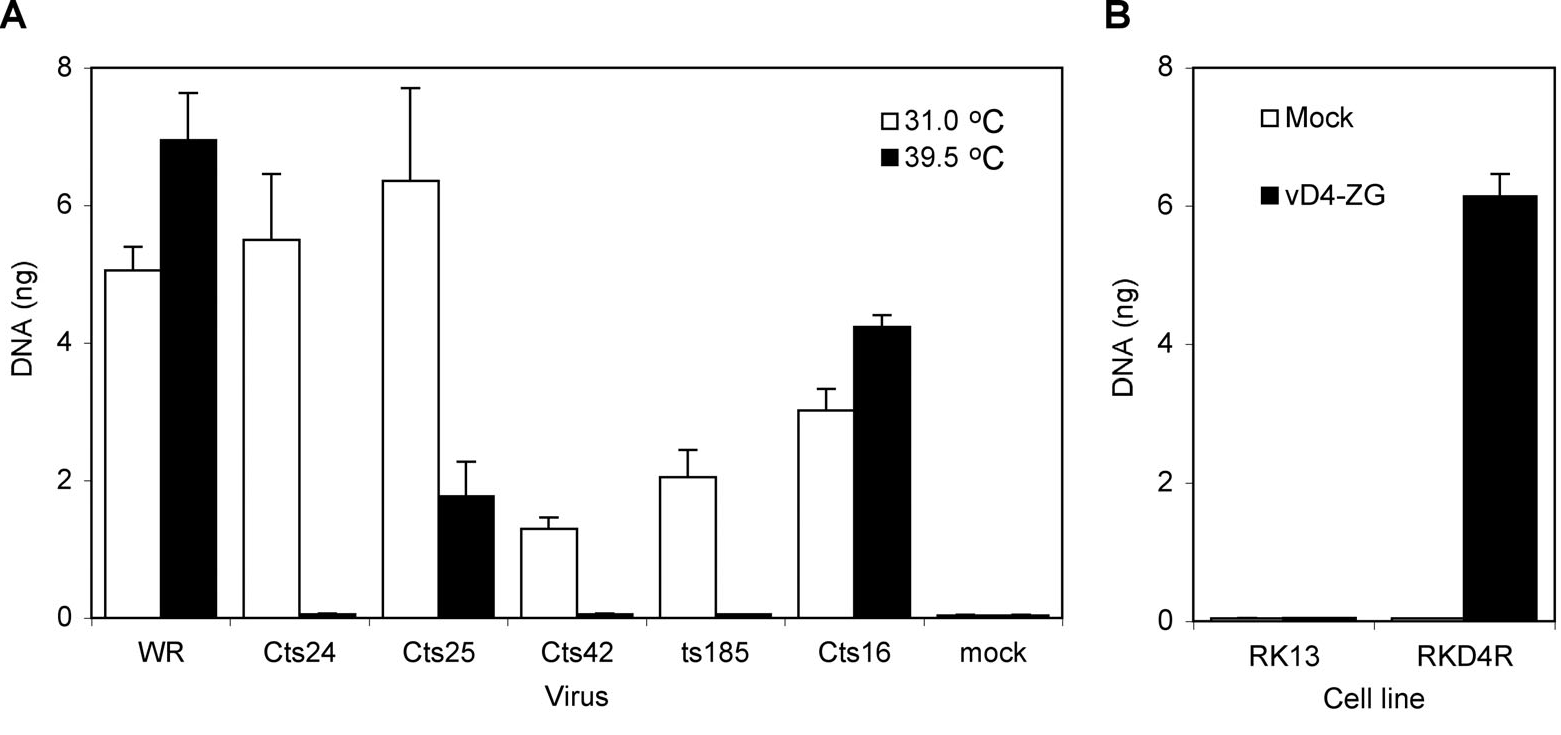
Viral protein requirements for plasmid replication. **(A) **Conditional lethal *ts *mutants. BS-C-40 cells were transfected with p716 and 24 h later were mock infected or infected with 3 PFU per cell of wild type VAC (WR) or *ts *mutant C*ts*24, C*ts*25, *ts*185, or C*ts*16 at permissive (31°C) and non-permissive (39.5°C) temperatures for 24 h. DNA was then isolated, digested with *Dpn*I and quantified by real-time PCR. **(B) **D4R deletion mutant. RKD4R and RK-13 cells were transfected with p716 and 24 h later were infected with 3 PFU of vD4-ZG. At 24 after infection, DNA was isolated, digested with *Dpn*I, and the amount of DNA quantified by real-time PCR.

The dependence of VAC genome replication on expression of five viral early proteins was previously determined by analysis of conditional lethal mutants. Because of the absence of *cis*-acting VAC DNA sequences, we considered that plasmid replication might only mimic DNA elongation steps and therefore require only a subset of viral proteins. To test this hypothesis, the plasmid was transfected into BS-C-40 cells (a derivative of BS-C-1 cells that have been passaged at 40°C), which were subsequently infected with a VAC *ts *mutant under permissive and non-permissive conditions. Plasmid replication was quantified by real-time PCR. Wild type VAC strain WR and C*ts*16, which has a mutation in the I7 gene encoding a protease required for VAC morphogenesis but not DNA synthesis [38], served as positive controls. Plasmid DNA synthesis was higher at 39.5°C than 31°C for both WR and C*ts*16 (Fig. 2A). In contrast, the reverse was true for each mutation known to impair DNA replication at the non-permissive temperature. Indeed, plasmid replication was barely detected at 39.5°C in cells infected with C*ts*24, C*ts*42, and *ts*185, which have defects in the D5 nucleoside triphosphatase, the E9 DNA polymerase, and the A20 processivity factor (Fig. 2A). The reduction in plasmid replication was less complete at 39.5°C in cells infected with C*ts*25, which has a defect in the B1 serine/threonine protein kinase, which is consistent with previous observations that showed viral genome accumulation was only moderately reduced in BS-C-40 cells at non-permissive temperatures [15]. The relatively low replication of plasmid at 31°C in cells infected with C*ts*42 and *ts*185 (Fig. 2A) suggested that the mutated DNA polymerase and processivity factor were still somewhat defective even under "permissive" conditions.

Previous studies had shown that expression of VAC uracil DNA glycosylase was required for genome replication [39]. To determine whether this protein is required for plasmid replication, we used mutant virus vD4-ZG, in which the uracil DNA glycosylase gene was deleted [39], and rabbit cell lines lacking (RK-13) or stably expressing (RKD4R) VAC uracil DNA glycosylase [39]. We found that plasmid DNA replication was only detected in the cell line stably expressing the viral uracil DNA glycosylase (Fig. 2B), indicating a requirement for this protein as well as each of the other four factors.

### Transfected plasmid DNA accumulates in viral factories

**Figure 3:**
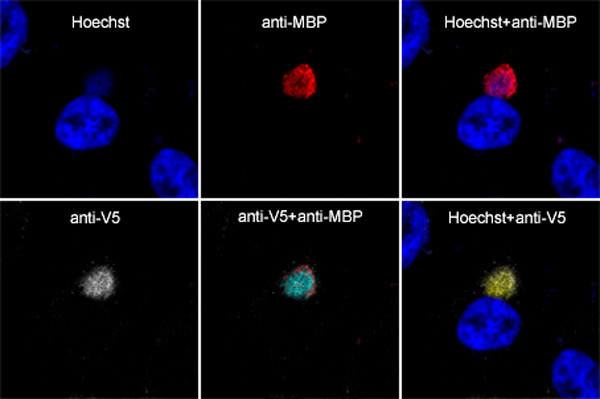
Intracellular localization of replicated plasmid containing tandem *lac*O repeats by staining with an MPB-*lac*I fusion protein. HeLa cells were transfected with pSV2-dhfr-8.32 containing *lac*O tandem repeats and 24 h later were infected with 3 PFU per cell of vV5D4 expressing V5-tagged uracil DNA glycosylase. At 12 h after infection, cells were fixed, permeabilized, incubated with MBP-*lac*I and rabbit antibody to MBP (anti-MBP) and mouse monoclonal antibody to the V5 epitope (anti-V5) followed by Cy5-conjugated donkey anti-mouse IgG and Texas red dye conjugated donkey anti-rabbit IgG. Cells were counterstained with Hoechst dye and analyzed by confocal microscopy. Colors: deep blue, Hoechst dye; red, Texas red; white, Cy5; light blue, overlap of Texas red and Cy5; yellow, overlap of Hoechst and Cy5.

VAC genomic DNA accumulates in specialized cytoplasmic factory areas near the nucleus. However, the intra-cytoplasmic location of plasmid replication had not been determined. We needed a specific tag to distinguish viral and plasmid DNA in order to locate the latter in infected cells. Several studies have used multimerized *E. coli lac *operator (*lac*O) binding sites and *lac *repressor (*lac*I) fusion protein interactions to examine chromatin organization and chromosome dynamics in living cells [40-43]. To apply this strategy, we transfected cells with a 10.5 kbp plasmid pSV2-dhfr-8.32 [44] containing 256 *lac*O repeats and infected the cells 24 h later. Initial experiments confirmed that plasmid replication occurred following VAC infection as described above for smaller plasmids (data not shown). Next we transfected cells with pSV2-dhfr-8.32 and then infected them with vV5D4, a recombinant VAC that expresses V5 epitope-tagged uracil DNA glycosylase. At 12 h after infection, DNA in the nucleus and cytoplasmic factories was visualized by Hoechst staining (Fig. 3). The plasmid appeared to be excluded from the nucleus and present exclusively in cytoplasmic viral factories as determined by staining the cells with a maltose binding protein (MBP)-*lac*I fusion protein and an antibody to MBP (Fig. 3). In addition, the plasmid sites contained the VAC DNA glycosylase, as shown by staining with antibody to the V5 tag of the latter protein (Fig. 3). No MBP staining was detected when a control plasmid lacking *lac*O sequences was transfected (data not shown).

### Visualization of replicating plasmid DNA in live cells

**Figure 4:**
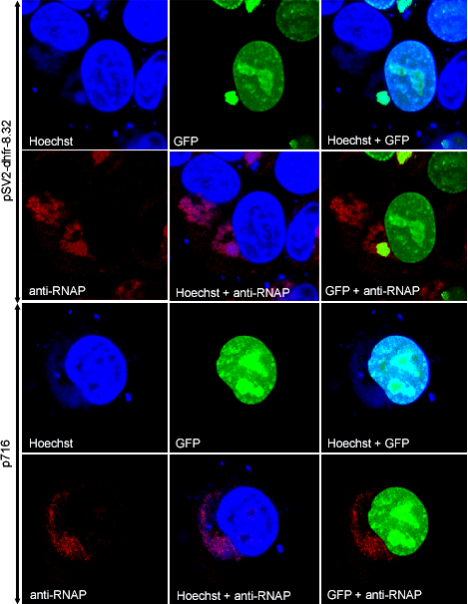
Intracellular localization of replicated plasmid containing tandem *lac*O repeats using a *lac*I-GFP fusion protein. HeLa cells were transfected with pSV2-dhfr-8.32 containing tandem *lac*O repeats (top 2 panels) or p716 control plasmid (bottom 2 panels) and infected with vGFP-*lac*I. At 12 h after infection, cells were fixed, permeabilized, and stained with antibody to VAC RNA polymerase (anti-RNAP), followed by Alexa 594-conjugated goat anti-rabbit IgG. Cells were then stained with Hoechst dye and analyzed by confocal microscopy. Blue, Hoechst; red, Alexa 594; and green, GFP fluorescence.

In the above experiment, the cells were fixed and stained in order to visualize the plasmid DNA. We considered that these steps might be avoided by expressing a GFP-*lac*I fusion protein with a nuclear localization signal (NLS). The GFP tag allowed visualization of *lac*I by fluorescence microscopy while the NLS served to translocate GFP-*lac*I, which was not specifically bound to *lac*O sequences in DNA, from the cytoplasm to the nucleus. In order to express the fusion protein prior to and during DNA replication, we constructed the recombinant vGFP-*lacI *with the open reading frame encoding the GFP-*lac*I-NLS fusion protein regulated by a viral early/late promoter. HeLa cells were transfected with pSV2-dhfr-8.32 and infected 24 h later with vGFP-*lacI*. Bright green fluorescence was detected over the viral factory areas and nuclei, which correlated with Hoechst staining (Fig. 4). In cells with multiple viral factories, however, not every one exhibited green fluorescence. The viral factory regions were also visualized by staining with an antibody to viral RNA polymerase, which surrounded and included the DNA sites at 12 h after infection (Fig. 4). When a control plasmid (p716) lacking *lac*O sites was transfected, the green fluorescence was strictly localized to the nucleus (Fig. 4).

**Figure 5:**
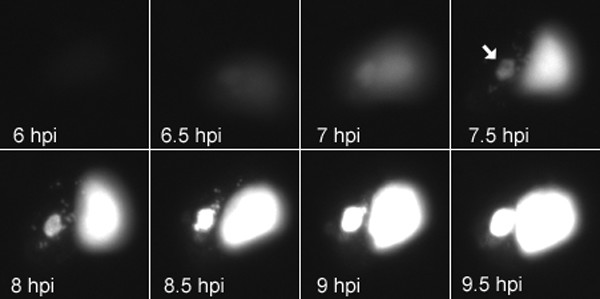
Visualization of plasmid replication in live HeLa cells. HeLa cells were transfected with pSV2-dhfr-8.32 containing tandem *lac*O repeats and infected with vGFP-*lac*I. Images were made at 5 min intervals starting at 5.5 h and ending at 10 h post infection. Selected images at the indicated time points are shown starting at 6 h time point. Arrow at 7.5 h time point indicates cytoplasmic site of replicated plasmid.

Having established the specificity of the GFP-*lac*I binding by co-localization, we examined fluorescence of live cells by time-lapse microscopy following transfection with pSV2-dhfr-8.32 and infection with vGFP-*lac*I. Weak GFP fluorescence was detected at about 5.5 h after infection (not shown) and was largely over the nucleus, reflecting the targeting due to the NLS. A region of juxtanuclear fluorescence corresponding to a viral factory was seen clearly at 7.5 h and over the next several hours increased in intensity (Fig. 5). The time course suggested that the factory region was the site of replication as well as accumulation of the plasmid DNA.

## Discussion

The replication of circular DNA lacking viral sequences as head-to-tail concatemers in the cytoplasm of cells infected with a poxvirus was reported nearly 20 years ago [29,30]. Fortuitous poxviral origins were ruled out by the replication of 5 different circular DNAs and no evidence was obtained for integration into the viral genome by non-homologous recombination. These data strongly suggested autonomous plasmid replication by a rolling circle or theta mechanism. The significance of sequence non-specific DNA replication was called into question by Du and Traktman [32], who reported only low-level replication of a super coiled plasmid compared to a linear minichromosome containing specific telomere sequences [32]. However, our determination of a 10-fold increase in net plasmid DNA compares favorably to the 2-fold increase achieved with the most efficient minichromosome construct [32]. Moreover, our finding was similar to the 8-fold increase in net plasmid DNA reported by DeLange and McFadden [30]. There are several procedural differences that might account for the disparate results. One difference was the type of virus and cell used: Du and Traktman used mouse L cells infected with VAC, DeLange and McFadden principally used rabbit cells infected with myxoma virus or Shope fibroma virus and we used monkey or HeLa cells infected with VAC. A second difference was the method of DNA isolation. Whereas we and DeLange and McFadden proteinase digested whole cell lysates, Du and Traktman lysed cells with cold hypotonic buffer containing a non-ionic detergent and removed nuclei by sedimentation prior to DNA extraction. VAC DNA replication occurs in juxtanuclear factories and loss of high molecular weight protein-DNA complexes, especially those containing long head-to-tail plasmid DNA concatemers upon centrifugation is a concern. Indeed, Du and Traktman [32] reported that the presence of the telomere resolution sequence was required for high efficiency replication of linear minichromosomes and that only monomeric products were recovered. Further studies are needed to determine whether the *cis*-acting sequences in the linear minichromosomes are serving as origins of replication or as concatemer resolution sites or both.

The temporal coincidence of plasmid and viral DNA replication suggested that viral proteins were needed for each. Indeed, we found that each of the five *trans*-acting viral proteins known to be important for viral genome replication was similarly required for plasmid replication. Either none of these proteins have a sequence-specific role or some have dual roles and are also required for origin-independent replication. The proteins also may have structural roles in assembling the replication complex, the existence of which is suggested by the interaction of A20 with the D4 and D5 proteins [45] and the co-purification of the A20, D4 and E9 proteins with a processive form of DNA polymerase [46,47].

VAC cores containing genomic DNA and an early transcription system travel from the cell entry site along microtubules to the juxtanuclear area where synthesis of early viral proteins and DNA replication result in the formation of discrete factories [48]. It is believed that each factory arises from a single infectious particle [49]. It was interesting to determine whether plasmid replication occurred in factories or dispersed throughout the cell. To investigate this, we transfected cells with a plasmid containing multiple repeats of the *E. coli lac*O, which tightly binds *lac*I. In one approach, the *lac*O DNA was located in discrete juxtanuclear regions by staining fixed and permeabilized cells with an MBP-*lac*I fusion protein followed by an antibody to MBP. The regions were identified as viral factories by Hoechst DNA staining and localization of the viral uracil DNA glycosylase, a protein required for replication of both plasmid and viral DNA. *Lac*O DNA was not detected in the nucleus or in diffuse areas of the cytoplasm. A second approach involved the construction of a recombinant VAC that expresses a GFP-*lac*I fusion protein with a NLS to remove unbound protein from the cytoplasm. Again, the *lac*O DNA was found in viral factories identified with Hoechst staining and viral RNA polymerase antibody. The data suggest that for plasmid replication to occur, the DNA must be at the right place i.e. a site containing viral replication proteins. Presumably the plasmid diffuses into the factory region and is captured by DNA binding proteins. By taking time lapse images of live cells, plasmid DNA was detected in juxtanuclear sites at 6 to 7 h after infection and increased in intensity as the factories enlarged over the next several hours. Factory enlargement appeared to occur from within rather than by fusion of multiple small factories. We suspect that the latter might occur if higher multiplicities of virus were used.

In contrast to the cytoplasmic replication of genome and plasmid DNA in VAC-infected cells, Sourvinos et al. [50] visualized nuclear replication of herpes simplex virus amplicons containing tetracycline operator sequence and Fraefel et al. [51] incorporated *lac*O sites into the genome of adenovirus associated virus and visualized discrete replication sites in the nucleus that fused to form larger structures. The latter study encouraged us to try to incorporate long tandem arrays of *lac*O repeats in the VAC genome, but they were unstable.

## Conclusion

We described a sensitive and quantitative real-time PCR method of measuring plasmid replication in cells infected with VAC and demonstrated that origin-independent replication requires all known viral replication proteins. In addition, we visualized the plasmid in living and fixed cells by incorporating tandem *lac*O sequences and determined that replication occurred in cytoplasmic viral factories. This system should be useful for studying the mechanism and minimal requirements of poxvirus DNA replication.

## Methods

### Cells, plasmids, and viruses

RK-13, BS-C-1, BS-C-40, HuTK^- ^143B, and HeLa cells were maintained in Eagle's minimal essential medium (EMEM; Quality Biologicals, Inc. Gaithersburg, MD) or Dulbecco's modified Eagle's medium (DMEM; Quality Biologicals, Inc.) containing 10% fetal bovine serum (FBS). A rabbit kidney cell line (RKD4R) stably expressing the VAC uracil DNA glycosylase and recombinant VAC vD4-ZG lacking a functional D4R gene [39] were gifts of F.G. Falkner. Plasmid pSV9 contains two copies of a 2.6 kbp insert derived from the VAC concatemer junction and two copies of pUC13 DNA [33]. Linear minichromosomes containing 1.3 kbp of VAC telomere sequences were prepared by ligation of snap cooled, *EcoR*I digested pSV9 essentially as described by Du and Traktman [32]. Ligation resulted in three products of 8 kbp, 2.6 kbp and 5.3 kbp. The 5.3 kbp minichromosome fragment was isolated by gel electrophoresis and the Qiaex II gel extraction kit (Qiagen). Plasmid p716 [52] was kindly provided by A. McBride; plasmids pSV2-dhfr-8.32 and p3'SS dimer-Cl-EGFP [44] were gifts of A. Belmont. The temperature sensitive (*ts*) replication mutants C*ts*16, C*ts*24, C*ts*42, C*ts*25 with mutations in the I7, D5, E9 and B1 open reading frames, respectively were obtained from R. Condit [53,54]; mut185 has a *ts *mutation in the A20 ORF [10].

### Antibodies

Cy5-conjugated affinipure F(ab')2 fragment of donkey anti-mouse IgG and Texas red dye conjugated affinipure F(ab')2 of donkey anti-rabbit IgG were obtained from Jackson ImmunoResearch laboratories. Alexa Fluor 594 goat anti-rabbit IgG was from Molecular probes. New England Biolabs and Invitrogen supplied the rabbit antibody to MBP and mouse anti-V5 monoclonal antibody, respectively.

### Transfection, infection and isolation of DNA

For experiments analyzed by real-time PCR, 0.1 μg of p716 DNA and 3.9 μg of salmon sperm carrier DNA were mixed with 10 μg of lipofectamine 2000 (Invitrogen) and uninfected cells were transfected according to the manufacturer's instructions. After 24 h, the cells were infected with VAC strain WR, vD4-ZG or a *ts *mutant at a multiplicity of 3 PFU per cell. Cells were then washed twice with Opti-MEM (Invitrogen) and overlaid with EMEM with 2.5% FBS. At various times, cells were harvested and the DNA isolated using the Qiamp DNA Blood Kit (Qiagen) according to the manufacturer's instructions. DNA was digested with restriction enzymes *Dpn*I or *Mbo*I (New England Biolabs).

### Southern blotting

DNA (2 μg) was digested with *EcoR*I and *Dpn*I or *Mbo*I, resolved on a 0.8% agarose gel, and transferred to Immobilon-Ny+ (Millipore) transfer membrane. Southern blotting was carried out as described by Maniatis [55]. Plasmid DNA was detected with a DNA probe that was ^32^P-labeled using a random-priming kit (Invitrogen). Pre-hybridizations and hybridizations were carried out using Quik-Hyb (Stratagene) according to the manufacturer's recommendation. The blot was exposed to a Phosphor screen and data acquired on a Storm 860 PhosphoImager (Molecular Dynamics, Sunnyvale, CA) and quantified with ImageQuant software (Molecular Dynamics).

### Real-time PCR

Oligonucleotides P1 (5'CAACTAAATGTGCAAGCAATGTAATTC3') and P2 (5'CATCCTGCCCCTTGCTGT3') were designed with Primer Express software supplied by Applied Biosystems. Reactions were carried out using SYBR Green PCR master mix (Applied Biosystems), 10 μM of each primer, and 1 ng of DNA in a total volume of 50 μl in an Applied Biosystems Prism 7900HT sequence detection system with v2.1.1 software. For amplification 40 cycles at 95°C for 15 s and 60°C for 60 s were used.

### Construction of recombinant viruses

vGFP-*lac*I: the open reading frame that encodes GFP-*lac*I was cloned by PCR using primers 5'CAGGCTGCGCAACTGTTGGGAAGGGCGA3' and 5'AAAAGTACTAGCCTGGGGTGCCTAATGAGTGAGC3' with p3'SS dimer-Cl-EGFP [44] as a template. The PCR product was digested with *Xho*I and *Sca*I and then ligated to *Xho*I and *Stu*I digested pSC59 [56] to form the plasmid pSC59gfp*lac*I. BS-C-1 cells were infected with VAC strain WR at 0.05 PFU per cell for 1 h and then transfected with 2 μg pSC59gfp*lac*I using 10 μg of Lipofectamine 2000. After 5 h, the medium was replaced with EMEM plus 2.5% FBS and the incubation continued for 2 days. Cells were harvested and lysed, and the diluted lysates were used to infect HuTK^- ^143B cell monolayers. The cells were overlaid with medium containing low melting point agarose and 25 μg of 5-bromodeoxyuridine per ml. After three rounds of plaque purification, the viral DNA was screened for the presence of the inserted DNA by PCR. The recombinant virus was propagated and titrated as described previously [57]. vV5D4: primers 5'ACTAGATACGTATAAAAAGGTATCTAATTTGATATAATGGGTAAGCCTATCCCTAACCCTCTCCTCGGTCTCGATTCTACGAATTCAGTGACTGT3' and 5'CTCCTGGACGTAGCCTTCGGG3' and DNA from plasmid pER-GFP [21] were used to add a V5 tag to the VAC D4R gene. After double digestion of the PCR product and plasmid with *Sna*BI and *Sma*I, the products were ligated together to form the new plasmid pERV5-GFP. Approximately 10^6 ^RKD4R cells were infected with vD4-ZG at a multiplicity of 0.05 PFU per cell for 1 h at 37°C. The infected cells were washed twice with Opti-MEM and transfected with 2 μg of pERV5-GFP using 10 μg of Lipofectamine 2000. After 5 h, the transfection mixture was replaced with EMEM containing 2.5% FBS, and the cells were harvested at 48 h in 0.5 ml of EMEM-2.5% FBS. Lysates were prepared by freezing and thawing the cells three times and sonicating them twice for 30 s. Recombinant viruses that expressed GFP were plaque purified five times on RKD4R cells. The genetic purity of recombinant viruses was confirmed by PCR and sequencing. The recombinant virus was propagated and titrated as described previously [57].

### Construction and expression of MBP-lacI

The *lac *repressor gene was PCR amplified using the following primers 5'CGGAATTCTCATCGGGAAACCTGTCGTGCCAGCTGC3' and 5'CGCGGATCCTAGTGAAACCAGTAACGTTATACG3' and template DNA from p3'SS dimer-Cl-EGFP. The amplified fragment was cloned into the *BamH*I and *Eco*RI sites of the expression vector pMal-c2x (New England Biolabs) resulting in the plasmid pMalc2x-*lac*I. Luria-Bertani medium (500 ml) supplemented with ampicillin (100 μg/ml) and glucose (0.2% w/v) was inoculated with 5 ml of an overnight culture of the *E. coli *ER2507 (New England Biolabs) containing the recombinant pMalc2x-*lacI *plasmid. The culture was grown at 37°C to a cell density of 0.5 at A600 nm and the expression of protein was induced for 2 h at 37°C by adding isopropyl-β-D-thiogalactopyranoside to a final concentration of 0.3 mM. The culture was then centrifuged at 4000 × *g *for 20 min at 4°C. A cell extract was prepared using B-PER reagent (Pierce) according to the manufacturer's recommendation and the protein purified using the pMAL protein fusion and purification kit (New England Biolabs).

### Confocal microscopy and live cell imaging

Cells were plated on glass cover slips in 12 well plates and transfected with 1 μg of pSV2-dhfr-8.32 using 5 μg of Lipofectamine 2000. After 24 h, cells were infected with recombinant VAC at 3 PFU per cell. At 12 h after infection, cells were fixed with cold 4% paraformaldehyde in phosphate buffered saline (PBS) at room temperature for 20 min. Fixed cells were permeabilized for 5 min with PBS containing either 0.2% Triton X-100 at room temperature. Permeabilized cells were incubated with primary antibodies at a 1:100 dilution in10% FBS for 30 min, washed with PBS three times, and then incubated with secondary antibody at a 1:100 dilution in 10% FBS for 30 min at room temperature. After washing with PBS three times, cover slips were incubated with Hoechst dye for 10 min at room temperature to visualize DNA staining. Stained cells were washed extensively with PBS and cover slips mounted in 20% glycerol. Cellular fluorescence was examined under a Leica TCS NT inverted confocal microscope and images were overlaid using Adobe Photoshop version 5.0.2.

For live cell imaging, HeLa cells were plated at ~80% confluence onto TC3 dishes (Bioptechs, Inc.) and infected with 3 PFU of virus per cell on the next day. Cells were imaged by either confocal or video microscopy. For video microscopy, a Hammumatsu C5985 camera and controller were attached to a Leica DMIRBE inverted fluorescence microscope. Images were digitized using an IC-PCI video capture card (Coreco Imaging, Inc.) controlled by Image Pro Plus software. Cells were maintained on a heated TC3 stage (Bioptechs, Inc.) with the temperature set at 37°C.

## Competing interests

The author(s) declare they have no competing interests.

## Authors' contributions

FDS participated in the design and coordination of the study, acquisition and analysis of data, and preparation of the manuscript. BM designed and coordinated the study, assisted in the data analyses and contributed to the preparation of the manuscript.
